# Home Non Invasive Ventilation (NIV) treatment for COPD patients with a history of NIV-treated exacerbation; a randomized, controlled, multi-center study

**DOI:** 10.1186/s12890-016-0184-6

**Published:** 2016-02-12

**Authors:** Kasper Linde Ankjærgaard, Philip Tønnesen, Lars Christian Laursen, Ejvind Frausing Hansen, Helle Frost Andreassen, Jon Torgny Wilcke

**Affiliations:** Department of Pulmonary Medicine, Gentofte Hospital, Kildegårdsvej 28, 2900 Hellerup, Denmark; Rigshospitalet Glostrup, The Danish Center of Sleep Medicine, Glostrup Nordre Ringvej 57, 2600 Glostup, Denmark; Department of Medicine, Herlev Hospital, Herlev Ringvej 75, 2730 Herlev, Denmark; Department of Pulmonary Medicine and Cardiology, Hvidovre Hospital, Kettegård Allé 30, 2650 Hvidovre, Denmark; Department of Pulmonary Medicine, Bispebjerg Hospital, Bispebjerg Bakke 23, 2400 København, NV Denmark

**Keywords:** COPD, Noninvasive ventilation (NIV), Acute hypercapnic respiratory failure, Exacerbations, Admissions, Randomized controlled trial

## Abstract

**Background:**

In chronic obstructive pulmonary disease, the prognosis for patients who have survived an episode of acute hypercapnic respiratory failure due to an exacerbation is poor. Despite being shown to improve survival and quality-of-life in stable patients with chronic hypercapnic respiratory failure, long-term noninvasive ventilation is controversial in unstable patients with frequent exacerbations, complicated by acute hypercapnic respiratory failure. In an uncontrolled group of patients with previous episodes of acute hypercapnic respiratory failure, treated with noninvasive ventilation, we have been able to reduce mortality and the number of repeat respiratory failure and readmissions by continuing the acute noninvasive ventilatory therapy as a long-term therapy.

**Methods:**

Multi-center open label randomized controlled trial of 150 patients having survived an admission with noninvasive ventilatory treatment of acute hypercapnic respiratory failure due chronic obstructive pulmonary disease. The included patients are randomized to usual care or to continuing the acute noninvasive ventilation as a long-term therapy, both with a one-year follow-up period. The primary endpoint is time to death or repeat acute hypercapnic respiratory failure; secondary endpoints are one-year mortality, number of readmissions and repeat acute hypercapnic respiratory failure, exacerbations, dyspnea, quality of life, sleep quality, lung function, and arterial gases.

**Discussion:**

Though previous studies of long-term noninvasive ventilation have shown conflicting results, we believe the treatment can reduce mortality and readmissions when applied in patients with previous need of acute ventilatory support, regardless of persistent hypercapnia.

**Trial registration:**

clinicaltrials.org: NCT01513655 16-Jan-2012.

## Background

Noninvasive ventilation (NIV) has been shown to improve survival in patients with chronic obstructive pulmonary disease (COPD), admitted with acute hypercapnic respiratory failure (AHRF) [1]; and NIV is recommended as a treatment of AHRF due to COPD [2]. COPD patients having survived AHRF have a poor prognosis; Chu et al. have shown one-year risks of readmission of 79.9 %; of a new life-threatening event (death or repeat AHRF) of 63.3 %; and of death of 49.1 % [3].

Hypothetically, long-term NIV (LTNIV) can improve the prognosis for severe COPD. The majority of trials have focused on patients with chronic hypercapnic respiratory failure due to stable COPD (i.e., patients with no exacerbations prior to LTNIV initiation); and their results have been conflicting [4]. However, in 2014, Köhnlein et al. published the results of a large randomized controlled trial (RCT) of LTNIV for stable patients with an arterial CO_2_ tension (*P*_a,CO2_) of 7 kPa or higher [5]: The patients treated with LTNIV had a one-year mortality of 12 % vs. 33 % for the controls; and the patients treated with LTNIV scored higher in health-related quality-of-life (HRQoL).

Three RCTs have studied the effects of LTNIV for unstable COPD, i.e., where the participants were enrolled after an admission with NIV treatment of AHRF: Cheung et al. and found that the patients treated with LTNIV had a lower rate of repeat AHRF (38.5 %/year vs. 60.2 %/year, *p* = 0.039) [6]; and Funk et al. found that the LTNIV group had a longer mean time to clinical worsening (391 days vs. 162 days; *p* = 0.0018) [7]. However, the largest RCT by Struik et al., showed no differences between the LTNIV group and the controls [8].

Little is known of what predicts AHRF in severe COPD. However, based on the large ECLIPSE study on exacerbations of COPD, Hurst et al. [9] and Müllerova et al. [10] showed that previous exacerbations predict new exacerbations [9]; and that previous admissions due to exacerbations of COPD predict new admissions and death [10].

We assume that previous admissions with AHRF due to COPD could predict new admissions with AHRF and need of NIV, as well as death.

We retrospectively analyzed 20 patients who were treated with LTNIV and followed at our pulmonary ward. The patients were offered LTNIV if they had been admitted for NIV treatment of AHRF at least twice. We found that the patients’ number of AHRF episodes and admissions due to COPD decreased significantly. And only four of the patients (20 %)—considerably less than the 49.1 % showed by Chu et al.—died within one year after initiation. Furthermore, these highly selected patients tolerated LTNIV well.

### Aim

The aim of this RCT is to investigate whether LTNIV can reduce mortality or repeat AHRF in patients who have been admitted for NIV treatment of AHRF due to COPD; and, secondarily, whether LTNIV can reduce mortality per se and the number of repeat AHRF, readmissions, and exacerbations, and improve quality of life.

## Methods/Design

COPD patients who are admitted for NIV treatment of AHRF (pH < 7.35 and *P*_a,CO2_ > 6 kPa) at Gentofte, Bispebjerg, Hvidovre or Herlev Hospitals in Greater Copenhagen are eligible. The patients must be optimally treated according to GOLD guidelines [2]. The patients must reside in the Capital Region, be able to give a verbal consent and sign a written consent form, and understand Danish.

The patients are excluded if they cannot comply with NIV or have a respiratory rate <12/min, severe hypoxia requiring more than 15 L O_2_/min, or a high risk of aspiration, recent abdominal, facial or upper airway surgery, malignancy or life expectancy of less than 6 months because of diseases other than COPD, known obstructive sleep apnea syndrome (OSAS), or a *St*HCO_3_^−^ less than 20 mmol/L.

### Design

The study is a multi-center randomized, controlled, open-label study. The participants are included during an admission with NIV treatment of AHRF due to COPD. If the patient gives consent (see below), he or she is randomized to either LTNIV or usual care. The acute NIV therapy is continued for as long the patient presents a hypercapnic acidosis or severe dyspnea without NIV; hereafter, NIV is ceased in the patients in the control group, whereas it is continued with the same settings in the LTNIV patients. When stable and when deemed appropriate by a physician, the patients are discharged.

The included patients will be given the possibility of contacting an acute hotline, operated by a nurse.

One week after discharge, all patients are visited by a nurse. The patients are followed in the outpatient departments with visits 1, 3, 6, 9, and 12 months after discharge.

A participant who is readmitted will not be re-randomized. If the participant is in the intervention group, LTNIV is resumed, if paused.

### Consent

When stabilized and able to have a small pause from NIV, the patient is presented to the study. If interested, the patient is formally informed of the study by one of the attending physicians. This is typically 2 days after admission, although individual. The information session is held with a physician who has devoted time for this and who is not disturbed. When informed, the consent form is signed by the patient and the informing physician. If the participant wants time for consideration, a new session will be planned one or two days later.

No individual data will be published.

### Intervention

For the intervention group, the acute NIV treatment is continued as LTNIV with the pressures and settings which had reversed the respiratory failure and the hypercapnic acidosis. The participant is offered to initiate an average volume-assured pressure support, but this is not mandatory.

Before discharge, the participant and caregivers are trained in handling and cleaning the ventilator, tubes, masks, etc. The participant is told to use the ventilator for a minimum of six hours of daily, preferably during sleep.

At the home visits and outpatient consultations, LTNIV is optimized according to the participant’s requests or complaints.

One month after discharge, LTNIV participants who have had only this one episode of AHRF and who do not feel a subjective improvement are offered to pause the LTNIV therapy, provided they have not had any of the following after discharge: exacerbation; need for antibiotics, increased oral corticosteroids, or inhaled medicine; hospitalization or visit to an emergency room or emergency physician due to COPD; fever of 38 °C or higher for at least one day; increased or purulent expectoration; or increasing dyspnea for at least one day.

If it is agreed to pause LTNIV, the participant is instructed to resume LTNIV at one or more of the above symptoms.

### Randomization

A total of 150 COPD patients will be included from the four wards. The participants are randomized to LTNIV or usual care in a 1:1 ratio, using a computer-generated block-randomization for each center. The participant is presented with a sealed envelope containing a piece of paper with either “A” (= LTNIV) or “B” (= usual care). The randomization list is stored at Gentofte Hospital in a sealed envelope. 50 sealed envelopes are prepared for each center.

### Outcomes

Primary outcome is difference in time to death *or* repeat AHRF between the study’s two groups. AHRF is defined as a pH < 7.35 and a *P*_a,CO2_ > 6 kPa despite optimal treatment according to GOLD guidelines, i.e., nebulized bronchodilators and intravenous corticosteroids [2].

The secondary outcomes are differences in the following variables; one-year mortality, admissions due to COPD; admissions due to repeat AHRF; visits to the emergency department or to an emergency physician due to COPD; exacerbations treated with oral corticosteroids or oral antibiotics; body mass index; forced expiratory volume in one second (FEV_1_); cost efficiency; health-related quality-of-life (HRQoL), measured by COPD Assessment Test (CAT) and Severe Respiratory Insufficience Questionnaire (SRI); sleep quality, measured by the Epworth Sleepiness Scale (ESS); apnea/hypopnea index (AHI); and Medical Research Council's Dyspnea scale (MRCD).

### Sample size/power calculation

Chu et al. showed that the one-year risk of death *or* repeat AHRF for COPD patients having survived an admission with NIV treatment of AHRF is 63.3 % [3]; and, for the LTNIV group in the RCT by Cheung et al., this risk was 38.5 % [6].

We use these risks for the power calculation. We accept a 0.05 risk of type 1 error (α) and 0.2 of type 2 error (β). With a power of 0.8, the needed sample size is 120. With an expected dropout of 20 %, we intend to include 150 participants, 75 in each arm.

### Measurements

Upon admission, we measure: arterial blood gases with *P*_a,CO2_, *P*_a,O2_, pH and *St*HCO_3_^−^, firstly to diagnose the AHRF and secondly, in order to titrate the NIV treatment; regular, venous blood samples, analyzing infectious parameters, red and white blood count, kidney- and liver parameters; electrocardiogram (ECG); and a chest x-ray.

These measurements are done as a standard at the acute admission and not as part of the protocol.

At discharge, we measure: lung function, i.e., FEV_1_, FVC and F_I_VC; peripheral oxygen saturation (*S*_pO2%_); arterial blood gases with *P*_a,CO2_, *P*_a,O2_, pH and *St*HCO_3_^−^; height and weight; MRC Dyspnea score; CAT, SRI and ESS questionnaires; and we note status of medication and long-term oxygen treatment (LTOT), when relevant.

For the intervention group, we read ventilator’s SIM card in regard to compliance, daily use, and apnea-hypopnea index (AHI).

At the home visit one week after discharge, we measure: lung function and *S*_pO2%_.

For the intervention group, we ensure that the ventilator, the mask and the hose are well-functioning; and that the patient is well informed and uses the ventilator correctly.

At the outpatient visits, we measure: lung function; *S*_pO2%_; arterial blood gases; weight; exhaled concentration of carbon mono-oxide; MRCD; CAT, SRI and ESS questionnaires; and we note status of medication, smoking, and LTOT, when relevant.

For the intervention group, we read ventilator’s SIM card in regard to compliance, daily use, and AHI.

At each outpatient visit and at the end of the patient’s study period, we collect data on mortality, hospital admissions, exacerbations treated by a general practitioner or an emergency physician, and emergency room visits.

### Collecting of data

All participants have an individual case report file (CRF) in which all data will be noted. The CRF contains nine sections; a front page with the basic information on the participant, i.e., the patient number, date of consent, allocation, ventilator settings (if relevant) etc.; and a section for each event in the study, i.e., admission, discharge, home visit, and the five outpatient visits including completion data in the fifth.

The CRF contains no personally identifiable information. The files are stored in a locked room to which only the investigators have access.

### Data processing

The primary outcome is the difference in time to death *or* AHRF between the LTNIV group and the control group, analyzed as an Intention-To-Treat analysis and calculated in a Cox proportional hazards regression, using Kaplan-Meier and log rank.

The differences between the study’s two groups will be computed using *X*^2^-statistics for the dichotomous variables and t-tests for the continuous variables, assuming that data will follow a normal distribution. Differences in time to a given event are calculated in a Cox proportional hazards regression, using Kaplan-Meier survival statistics and log rank.

For all comparisons, *p* < 0.05 is the level of significance.

For the sample size calculation, we used SAS (Statistical Analysis System, version 9.4).

We will use the latest versions of SAS for the statistical analyses.

### Publications

Both positive and negative results will be published in national and international journals and at conferences, symposia, etc.

### Ethics

The study was approved by the Regional Committee on Health Research Ethics (DNVK) of The Capital Region, Data Protection, and Drug Authority. Trial no. H-1-2011-084.

The trial is conducted at four different geographical locations:

1. Dept. of Pulmonary Medicine Y, UH Gentofte, Hellerup, Denmark, DK-2900
2. Dept. of Internal Medicine O, UH Herlev, Herlev, Denmark, DK-2730
3. Dept. of Pulmonary Medicine and Cardiology, UH Hvidovre, Hvidovre, Denmark, DK-2650
4. Dept. of Pulmonary Medicine, L, UH Bispebjerg, København NV, Denmark, DK-2400

The four hospitals are all governed by the Capital Region of Denmark; thus, administratively and legally, the four hospitals are one. Therefore, by Danish law, only one ethics committee approvalwas needed.

Trial registration:

The trial was registered at clinicaltrials.org. Registration number: NCT01513655, 16-Jan-2012.

No individual or personally identifiable data will be published.

### Biological material

The only biological samples taken are general blood biochemistry and arterial blood gases. This is the standard for any admission and outpatient visit. The samples are handled by the Dept. of Clinical Biochemistry according to current guidelines; after analysis, the samples are destroyed. No biological material is stored. Arterial blood gas samples measure approximately 1 mL; venous blood samples measure approximately 10–15 mL.

## Discussion

This RCT will assess whether LTNIV can reduce mortality and prevent exacerbations and repeat AHRF in unstable COPD patients having survived an admission due to AHRF. Three previous RCTs have assessed LTNIV for COPD patients after an episode of AHRF, treated with NIV; the results have been conflicting as the two smaller RCTs showed that LTNIV could reduce repeat AHRF and clinical worsening [6, 7], whereas the largest RCT showed no effect [8].

In the large RCT and in one of the two small, an additional inclusion criterion was persistent hypercapnia without acidosis after the AHRF episode [7, 8].

It is documented that previous exacerbations of COPD predict new exacerbations [9] and that previous admissions due to exacerbations predict new admissions and death [10].

Based on this and on our experience with LTNIV for COPD, we believe that LTNIV can reduce mortality and repeat AHRF, regardless of whether the patient was persistently hypercapnic after the acute NIV treatment. We are looking forward to assessing this in an RCT.

### Blinding

The study is randomized with an intervention group and a control group. The random sequence generation is random and unknown to the investigators. However, the study is open label, i.e., unblinded; the patient allocation cannot be concealed, as the patients will necessarily know whether they receive LTNIV or usual care. We considered the possibility of using sham NIV or CPAP in the control group; but we assume it would not be worthwhile, as the patients would undoubtedly figure out their allocation. Furthermore, CPAP could affect quality-of-life negatively and thus favor the NIV treatment group. We believe that—by offering the same outpatient monitoring in the two arms, and as our primary outcome is mortality—the risk of bias in an open trial will be minimal.

### Risks

There are few side effects of NIV treatment:

Some people feel uncomfortable wearing the mask, as it fits tightly to the face; some will experience dryness of the eyes, nose and mouth, due to the airflow. The mask can—if not fitted and placed properly—give pressure ulcers, especially over the nose. Due the high ventilator pressures, the user can get a sense of flatulence. NIV should not be used if the user tends to vomit because of the risk of aspiration. The patients are thoroughly instructed orally and in writing to use the NIV in a semi-sitting position (45°).

The risks of blood samples are negligible and limited to the risk of hematoma at the site of injection.

Besides these minor risks, we believe that participation in the present RCT is without risks.

## Abbreviations

NIV: noninvasive positive pressure ventilation
COPD: chronic obstructive pulmonary disease
AHRF: acute hypercapnic respiratory failure
LTNIV: long-term noninvasive ventilation
CHRF: chronic hypercapnic respiratory failure
RCT: randomized controlled trial
*P*_a,CO2_: arterial partial pressure of carbon dioxide
HRQoL: health-related quality-of-life
*S*_pO2%_: peripheral oxygen saturation
FEV_1_: forced expiratory volume in one second
FVC: forced vital capacity
F_I_VC: forced inspiratory vital capacity
GOLD: Global initiative for chronic Obstructive Lung Disease
OSAS: obstructive sleep apnea syndrome
AVAPS: average volume-assured pressure support
CAT: COPD Assessment Test
SRI: Severe Respiratory Insufficience questionnaire
ESS: Epworth Sleepiness Scale
MRCD: Medical Research Council Dyspnea score
AHI: Apnea/Hypopnea Index
*P*_a,O2_: arterial partial pressure of oxygen
LTOT: long-term oxygen therapy
CRF: case report file
